# Are village health volunteers as good as basic health staffs in providing malaria care? A country wide analysis from Myanmar, 2015

**DOI:** 10.1186/s12936-018-2384-4

**Published:** 2018-06-20

**Authors:** Nay Yi Yi Linn, Soundappan Kathirvel, Mrinalini Das, Badri Thapa, Md. Mushfiqur Rahman, Thae Maung Maung, Aye Mon Mon Kyaw, Aung Thi, Zaw Lin

**Affiliations:** 1 National Malaria Control Programme, Department of Public Health, Ministry of Health and Sports, Nay Pyi Taw, Myanmar; 2 The International Union of Tuberculosis and Lung Diseases, Union South-East Asia Regional Office, New Delhi, India; 3 Department of Community Medicine, School of Public Health, Postgraduate Institute of Medical Education and Research, Chandigarh, India; 4 Médecins Sans Frontières-OCB, New Delhi, India; 5 World Health Organization Country Office for Myanmar, Yangon, Myanmar; 6 Department of Medical Research, Ministry of Health and Sports, Nay Pyi Taw, Myanmar

**Keywords:** Health workforce, Accessibility, Quality of care, Community health worker, Performance, Greater Mekong Sub-region

## Abstract

**Background:**

Malaria is one of the major public health problems in Myanmar. Village health volunteers (VHV) are the key malaria diagnosis and treatment service provider at community level in addition to basic health staffs (BHS). This countrywide analysis aimed to assess and compare the accessibility to- and quality of malaria care (treatment initiation, treatment within 24 h and complete treatment delivery) between VHV and BHS in Myanmar.

**Methods:**

This was a retrospective cohort study using record review of routinely collected programme data available in electronic format. All patients with undifferentiated fever screened and diagnosed for malaria in January–December 2015 by VHV and BHS under National Malaria Control Programme in Myanmar were included in the study. Unadjusted and adjusted prevalence ratios (aPR) were calculated to assess the effect of VHV/BHS on receipt of treatment by patients.

**Results:**

Of 978,735 undifferentiated fever patients screened in 2015, 11.0% of patients were found malaria positive and the malaria positivity in VHV and BHS group were 11.1 and 10.9% respectively. Access to malaria care: higher proportion of children aged 5–14 years (21.8% vs 17.3%) and females (43.7% vs 41.8%) with fever were screened for malaria by VHV compared to BHS. However, the same for children aged < 5 years was 2.2% lower in VHV group compared to BHS. Quality of malaria care: the proportion of malaria cases that received treatment was 96.6 and 94.9; treatment initiation within 24 h of fever was 44.7 and 34.1; and, complete treatment delivery was 80.9 and 88.2, respectively, in VHV and BHS groups. After adjustment for potential confounders, patients with malaria provided care by VHV had 1.02 times higher chance of receiving treatment compared to BHS [aPR (95% confidence interval) 1.017 (1.015, 1.020)].

**Conclusions:**

The VHV were more accessible to children and women than BHS in providing malaria screening services. The malaria treatment services provided by VHV was as good as BHS. Further qualitative research to explore and address the challenges on initiation and delivering complete treatment by VHV including inventory assessment and cost-effectiveness studies on integration of VHV in routine health system are needed.

## Background

Myanmar is one the malaria endemic countries in the South-east Asia Region (SEAR) aiming to eliminate malaria by 2030 according to National Strategic Plan for malaria elimination [1]. The country once reported highest mortality (53.6%) due to malaria in the region even though it shared only 7% of total cases in the region [2]. The mortality was high among population residing in Thai and Indian borders, internally displaced population in conflict zones and among ethnic minority regions [3, 4]. The country also noted as an epicentre for drug resistant malaria due to incomplete and inappropriate use of anti-malarial drugs, use of fake or expired anti-malarial drugs and expensive artemisinin derivatives [3].

Despite the political crisis and conflicts, difficult geographic area and related access, spreading drug resistance in the country, it was reported that the country has reduced the malaria morbidity by 81.1% and mortality by 93.5% from 2005 to 2014 [5]. Similarly, the high malaria transmission area (> 1 case per 1000 population) has also been reduced from 53 to 16% [6, 7]. *Plasmodium falciparum* is always the most commonest infection in Myanmar which contributes to 65–70% of cases followed by *Plasmodium vivax* [7]. The key interventions quoted for the successful reduction of malaria burden were placement of village health volunteers (VHV) strategically at rural, remote, hard to reach and conflict areas, good coverage of insecticide-treated bed nets among at-risk population and improved access to artemisinin-based combination treatment [5, 8].

Based on the evidence created through government and national/International non-government organizations (NGOs), Myanmar introduced VHV in 2007 at community level to improve the access and achieve universal coverage of malaria prevention and care services among rural and hard to reach population [9–12]. A total of 40,000 VHV (half under national malaria control programme and half under various NGOs) are trained in the country, of which 15,000 are actively providing services related to malaria prevention and care [13]. In addition to early diagnosis of malaria using rapid diagnostic kit test (RDT) and delivery of first-line anti-malarial drugs as per national malaria treatment guidelines, they also deliver the insecticide-treated bed nets, and provide malaria information and advice to at-risk population [10, 13].

VHV are trained and supervised by basic health staffs (BHS-namely health assistant, lady health visitor, auxiliary nurse midwives, and public health supervisors) placed at different types of public health facilities like township hospitals, station hospitals, rural health centres (RHCs) and sub-RHCs. BHS deliver clinic and home based preventive, promotive and curative (treatment and referral) healthcare services related to all national health programmes namely maternal and child health (antenatal and postnatal care, immunization, contraception), communicable disease including vector borne disease surveillance and control, school health, treatment of minor illnesses like diarrhoea and acute respiratory infection, and others [12, 14]. VHV are recruited to complement the malaria control activities of BHS at rural, hard to reach population.

A dedicated workforce like placement of VHV was successful in improving the access to and utilization of healthcare services related malaria in Cambodia, Zambia, Ghana, and other countries [12, 15–20]. However, a systematic review found insufficient evidence to comment on effectiveness of malaria control interventions by VHV in reducing morbidity and mortality [21]. Further, studies comparing the performance of VHV with existing formal healthcare workers in delivering malaria control activities are limited. As Myanmar is planning to recruit more VHV and upgrade them as integrated community malaria volunteers (additional responsibilities to provide dengue, lymphatic filariasis, HIV, tuberculosis, leprosy services) in the near future, it is time to review their performance in delivering malaria control activities before providing additional responsibilities.

This countrywide study was conducted to assess and compare the malaria diagnostic and treatment services provided by VHV and BHS under National Malaria Control Programme (NMCP) of Myanmar in 2015. The specific objectives of the study were to determine and compare VHV and BHS by: (a) number and proportion of patients screened for malaria; (b) number and proportion of patients diagnosed with malaria; (c) number and proportion of patients with malaria initiated on treatment; (d) number and proportion of patients with malaria initiated on treatment within 24 h of fever; (e) number and proportion of patients with malaria provided complete treatment; and, (f) to assess the influence of type of healthcare provider on treatment initiation after adjustment with demographic and clinical factors.

## Methods

### Study design

This was a retrospective cohort study using record reviews from routinely collected programme data of NMCP of Myanmar in 2015.

### Study setting

Myanmar is a lower middle income country, bordered by China on the north and north-east, India and Bangladesh on the west, Laos and Thailand on the east and south-east, and Andaman sea and Bay of Bengal on the south. The country consists of 14 region/states and Nay Pyi Taw, a union council territory, with a population of 51.5 million [22]. The vector-borne disease control (VBDC) programme of the country is responsible for delivering integrated healthcare services on prevention and control of malaria, dengue, zika, chikungunya, Japanese encephalitis, and lymphatic filariasis. The NMCP has adopted a three-pronged approach to provide malaria test-treat-track services to populations at risk [1].

### VHV and BHS

**Fig. 1:**
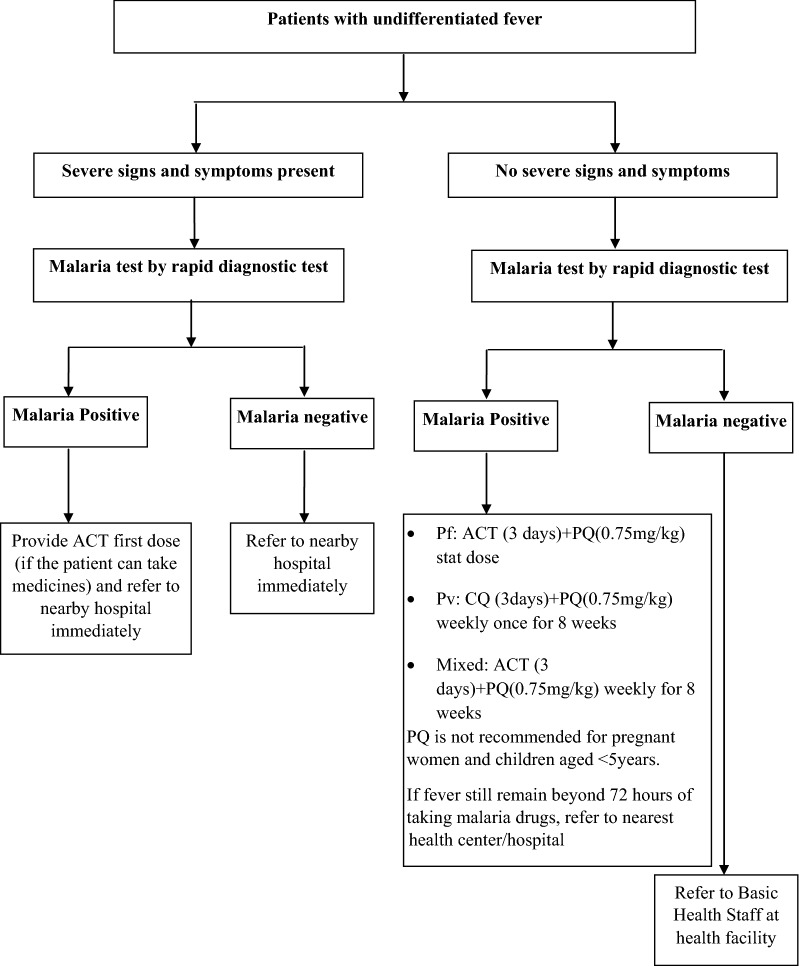
The algorithm of malaria screening, diagnosis and treatment services provided by village health volunteers. Pf: *Plasmodium falciparum*; Pv: *Plasmodium vivax*; mixed: *Plasmodium falciparum* and *Plasmodium vivax*; ACT: Artemisinin based combination therapy (Artemether + Lumefantrine); PQ: Primaquine; CQ: Chloroquine; RDT: rapid diagnostic test with dual antigen

VHV are the key service providers in Myanmar at village/community level in delivering malaria diagnosis and treatment services. They are recruited and trained by NMCP and other implementing national and international NGOs. VHV working under the NMCP receive 5 days modular training on malaria diagnosis and treatment. The training of VHV is provided by the BHS of the respective area. VHV use rapid diagnostic test (RDT)-dual antigen (*P. falciparum* and *P. vivax*) for testing and initiating treatment, if found positive, according to national malaria treatment guidelines (NMTG) [1]. The algorithm of screening, testing and management of malaria by VHV is given in Fig. 1. As per the NMTG, VHV should refer all pregnant women and children less than 1 year with malaria, and complicated malaria to BHS of higher health facility for further treatment [12]. VHV receive 21,000–50,000 kyats (USD 15.5 to 36.9) as incentive every 3 months to support their travel related to malaria activities [13].

**Fig. 2:**
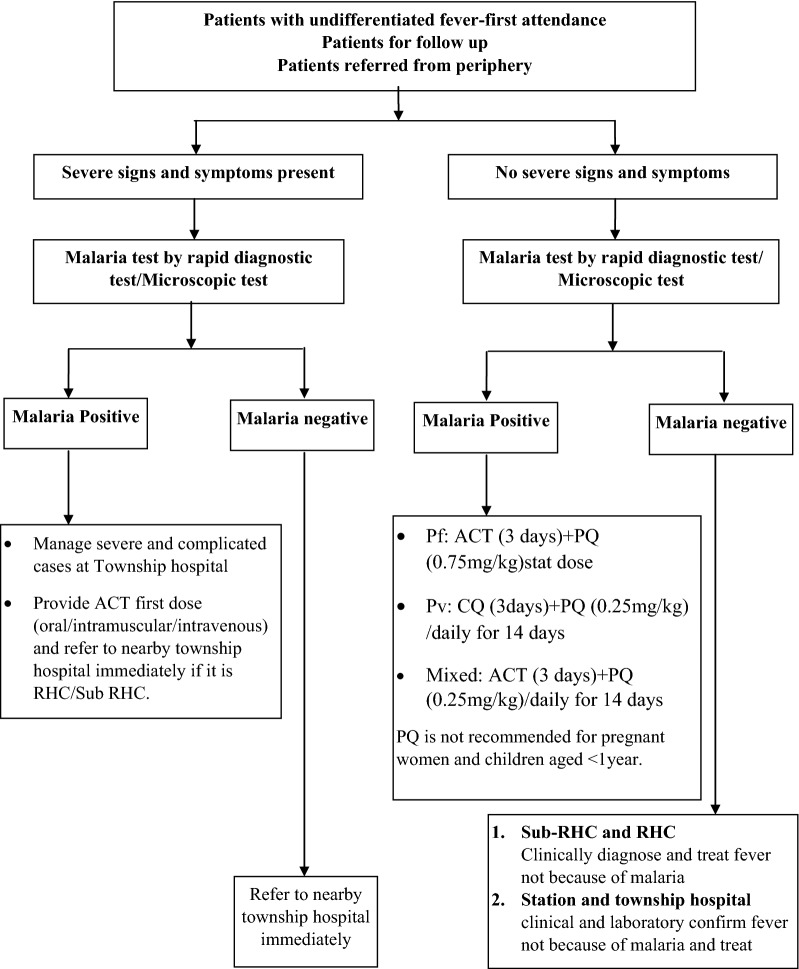
The algorithm of malaria screening, diagnosis and treatment services provided by basic health staffs. Pf: *Plasmodium falciparum*; Pv: *Plasmodium vivax*; mixed: *Plasmodium falciparum* and *Plasmodium vivax*; ACT: Artemisinin based combination therapy (Artemether + Lumefantrine); PQ: Primaquine; CQ: Chloroquine; RDT: rapid diagnostic test with dual antigen; RHC-Rural health centre

Malaria control services are provided by township health departments, which include a township hospital, 1–3 station health unit/hospitals, 4–5 rural health centres (RHCs) and 4–5 sub-RHCs per station hospital and RHC. Each township hospital is manned with medical officers to manage the hospital, maternal and child health and school health, and team leaders for tuberculosis, leprosy, trachoma, sexually transmitted diseases, and vector borne disease control. The township hospital is equipped with extended laboratory services other than microscopic examination of blood smear and with the facilities to manage severe and complicated malaria cases. Each station hospital manned with a medical officer and 1–2 health assistants. Each station hospital caters to 4–5 RHCs. A RHC should have a group of 12 BHS: 1 health assistant, 1 health visitor, 5 midwives, and 5 public health supervisors as per Ministry of Health and Sports guidelines. The majority of RHCs have vacant positions and are manned with only one midwife and one public health supervisor. Each sub-RHCs is manned with a midwife [1, 13]. There are around 12,000 public health facilities available throughout the country and around 30,000 BHS are working in these facilities [14, 22, 23]. The algorithm of screening, testing and management of malaria by BHS is given in Fig. 2.

### Malaria case screening, treatment and reporting

VHV and BHS screen all patients with fever for malaria and initiate treatment according to NMTG (Figs. 1 and 2). The duration and schedule of primaquine is the main difference between VHV (weekly once for 8 weeks) and BHS (daily for 14 days) group in case of *P. vivax* or mixed infection. Similarly, primaquine is not delivered to ≤ 5 years children by VHV and ≤ 1 year children by BHS. They deliver the complete course of first line anti-malarial drugs during delivery of first dose as part of multi-tablet blister packs (different blister pack for different age groups). The operational definitions related to completeness of treatment are given in Box 1 [24]. Details of the patients screened, diagnosed and treated are entered into a structured carbonless register by VHV and BHS and sent to the township VBDC office every month. The data assistant at township or state/regional level enters the data in the national malaria compile database (Microsoft Excel-based).

### Box 1: Operational definitions for completeness of malaria treatment provided in Myanmar 2015 [19]

**Table:** 

1. Complete treatment	Treatment of malaria with appropriate schizonticidal and gametocidal drugs in adequate dose and duration appropriate to species, age and pregnancy status of the patients as given in national malaria treatment guidelines. The duration is calculated as delivery of complete course of drugs for adequate duration along with first dose of antimalarial drugs as there is no follow up done after delivery of first dose of anti-malarial drugs
2. Incomplete treatment	Partial course (dose or duration) of either schizonticidal drugs or gametocidal drugs or both. Treatment with appropriate schizonticidal drugs (full course) according to malaria species but not gametocidal drugs as per national malaria treatment guidelines
3. Inappropriate treatment	Treatment with schizonticidal and gametocidal drugs not in line with malaria species as per national malaria treatment guidelines

### Study population and period

All patients diagnosed with undifferentiated fever screened for malaria between January 2015 and December 2015 by VHV and BHS under NMCP in Myanmar were included in the study.

### Data variables and sources of data

The variables routinely collected in the carbonless register are: age, gender, pregnancy status, type of healthcare provider, test result, species type, severity of malaria, treatment initiation status, time of initiation of treatment (≤ 24 or > 24 h of fever) and the regimen of treatment provided. As per the NMTG (Fig. 1) and operational definition Box (1), the completeness of treatment has been classified.

### Analysis and statistics

The electronic data routinely entered in the national malaria compile database (Microsoft Excel-based) was exported to STATA (version 11.0, copyright 1984–2009, Stata Corp, Texas, USA, Serial Number: 30110549055) for descriptive and inferential analysis. Number and proportions were calculated to describe the demographic, clinical and treatment characteristics of patients with malaria. Chi squared test was used to check variability of demographic, clinical and treatment characteristics of patients screened and treated for malaria with the type of healthcare provider. Unadjusted and adjusted analyses were carried out to assess the influence of type of healthcare provider on treatment initiation. Factors with p < 0.2 in unadjusted analysis were included to calculate the adjusted prevalence ratio-aPR. Severity of the malaria was removed from the model as complicated malaria cases were very less and were supposed to be referred to higher health facility for management. A p value of < 0.05 was considered statistically significant.

## Results

### Characteristics of patients with undifferentiated fever screened for malaria

A total of 978,735 patients with undifferentiated fever were screened for malaria under NMCP of Myanmar in 2015. Of which, 27.6% were screened by VHV and 72.4% were screened by BHS. Among those screened, 9.5% were children aged < 5 years and 42.3% were females. Around 1.5% of females were reported to be pregnant. The demographic characteristics of patients with undifferentiated fever screened for malaria by VHV and BHS is given in Table 1.

**Table 1:** Demographic characteristics of patients with undifferentiated fever tested for malaria by VHV and BHS in Myanmar, 2015

Characteristics	Total	VHV	BHS	p value^c^
	n (%)	n (%)	n (%)	
Total^a^	978,735 (100)	270,155 (27.6)	708,580 (72.4)	
Age (years)				
< 5	92,561 (9.5)	21,266 (7.9)	71,295 (10.1)	< 0.001
5–14	181,834 (18.6)	58,906 (21.8)	122,928 (17.3)	
≥ 15	704,340 (72.0)	189,983 (70.3)	514,357 (72.6)	
Sex				
Male	564,619 (57.7)	152,010 (56.3)	412,609 (58.2)	< 0.001
Female	414,116 (42.3)	118,145 (43.7)	295,971 (41.8)	
Pregnancy status^b^				
Yes	5644 (1.5)	1044 (1.0)	4600 (1.7)	< 0.001
No	374,076 (98.5)	106,757 (99.0)	267,319 (98.3)	

All are column percentages except ^a^ which is row percentage; *VHV* Village health volunteers, *BHS* Basic health staffs; ^b^ pregnancy status is missing among 34,374 females (VHV-10325 and BHS-24049); ^c^ chi-squared test was used

### Demographic, clinical and treatment characteristics of patients diagnosed with malaria

**Fig. 3:**
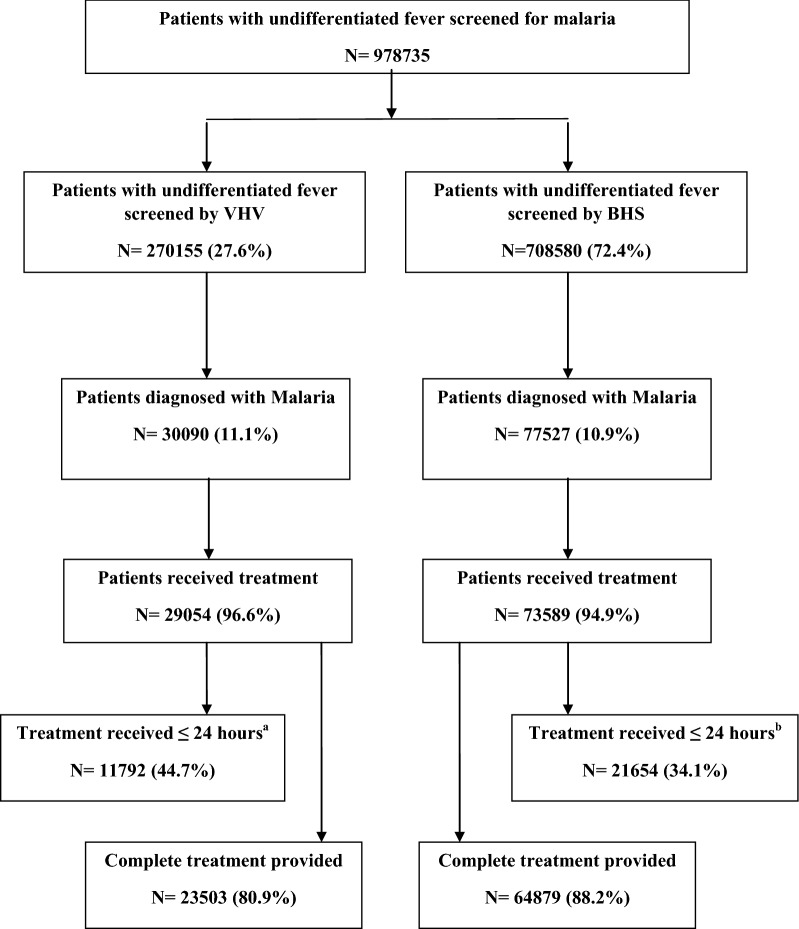
Cascade of diagnosis and treatment services provided by VHV and BHS for patients with Malaria in Myanmar, 2015

About 11.0% of patients with undifferentiated fever were found malaria positive. The malaria positivity was higher in patient aged ≥ 15 years (11.3%) and in males (12.9%) compared to other groups (Table 2). Of 107,617 patients with malaria, 8.3% were < 5 years and 32.4% were females. Among females with malaria, 1.5% were pregnant. *Plasmodium falciparum* either alone or mixed with *P. vivax* was found in 68.3% of cases. In total, 95.4% patients with malaria had received treatment. Among treated, 86.1% were given complete treatment. The demographic, clinical and treatment characteristics of malaria cases provided by VHV and BHS are described in Table 3. The cascade of malaria screening and treatment services provided under NMCP of Myanmar is given in Fig. 3.

**Table 2:** Results of malaria test done by VHV and BHS among patient with undifferentiated fever in Myanmar, 2015

Characteristics	Total		VHV		BHS		p value^b^
	Number tested	Test positive	Number tested	Test positive	Number tested	Test positive	
	n	n (%)	n	n (%)	n	n (%)	
Total	978,735	107,617 (11.0)	270,155	30,090 (11.1)	708,580	77,527 (10.9)	0.005
Age (years)							
< 5	92,561	8917 (9.6)	21,266	3092 (14.5)	71,295	5825 (8.2)	< 0.001
5–14	181,834	19,324 (10.6)	58,906	6989 (11.9)	122,928	12,335 (10.0)	< 0.001
≥ 15	704,340	79,376 (11.3)	189,983	20,009 (10.5)	514,357	59,367 (11.5)	< 0.001
Sex							
Male	564,619	72,716 (12.9)	152,010	19,574 (12.9)	412,609	53,142 (12.9)	0.978
Female	414,116	34,901 (8.4)	118,145	10,516 (8.9)	295,971	24,385 (8.2)	< 0.001
Pregnancy^a^							
Yes	5644	527 (9.3)	1068	191 (18.1)	4613	336 (7.3)	< 0.001

All are row percentages

*VHV* Village health volunteers, *BHS* Basic health staffs

^a^ Pregnancy status is missing among 34,374 females (VHV-10325 and BHS-24049)

^b^ Chi-squared test compared the malaria positivity between VHV and BHS

**Table 3:** Demographic, clinical and treatment characteristics of patients with malaria diagnosed under NMCP by VHV and BHS in Myanmar, 2015

Characteristics	Total n (%)	VHV n (%)	BHS n (%)	p value^f^
Total^a^	107,617 (100)	30,090 (28.0)	77,527 (72.0)	
Age (years)				
< 5	8917 (8.3)	3092 (10.3)	5825 (7.5)	< 0.01
5–14	19,324 (18.0)	6989 (23.2)	12,335 (15.9)	
≥ 15	79,376 (73.8)	20,009 (66.5)	59,367 (76.6)	
Sex				
Male	72,716 (67.6)	19,574 (65.1)	53,142 (68.5)	< 0.01
Female	34,901 (32.4)	10,516 (34.9)	24,385 (31.5)	
Malaria species^b^				
Pf	70,256 (65.3)	20,797 (69.1)	49,459 (63.8)	< 0.01
Pv	34,145 (31.7)	7934 (26.4)	26,211 (33.8)	
Mixed	3210 (3.0)	1359 (4.5)	1851 (2.4)	
Severity^c^				
Complicated	1340 (1.3)	383 (1.3)	957 (1.3)	0.38
Uncomplicated	101,999 (98.7)	28,049 (98.7)	73,950 (98.7)	
Treatment status				
Treated	102,643 (95.4)	29,054 (96.6)	73,589 (94.9)	< 0.01
Not treated	4974 (4.6)	1036 (3.4)	3938 (5.1)	
Time of treatment (h)^d^				
≤ 24	33,446 (37.2)	11,792 (44.7)	21,654 (34.1)	< 0.01
> 24	56,497 (62.8)	14,615 (55.3)	41,882 (65.9)	
Treatment provided^e^				
Complete	88,382 (86.1)	23,503 (80.9)	64,879 (88.2)	< 0.01
Incomplete/inappropriate	14,261 (13.9)	5551 (19.1)	8710 (11.8)	

All are column percentages except ^a^ which is row percentage; VHV: Village health volunteers; BHS: Basic health staffs; Pf: *Plasmodium falciparum*; Pv: *Plasmodium vivax*; mixed: Both Pf and Pv positive; ^b^ 6 cases of Plasmodium malariae and ovale omitted from the analysis; ^c^ 4278 cases missing severity; ^d^ 12,700 cases missing time of treatment among treated; ^e^ 4974 cases did not receive treatment; ^f^ chi-squared test has been used

### Comparison of performance of VHV and BHS

#### Access to malaria screening services among patients with undifferentiated fever

VHV provided malaria-screening services to a higher proportion of children aged 5–14 years (21.8% vs 17.3%) and females (43.7% vs 41.8%) with undifferentiated fever compared to BHS. However, the proportion of children aged < 5 years with undifferentiated fever screened for malaria by VHV (7.9%) was 2.2% lower than BHS (10.1%) (Table 1). Though VHV screened high proportion of females compared to BHS, the proportion of pregnant females screened was lower (1.0% vs 1.7%) in VHV group compared to BHS.

The overall malaria positivity was 11.1% and 10.9% respectively among patients screened by VHV and BHS. The malaria positivity among children aged < 5 years (14.5% vs 8.2%), children aged 5–14 years (11.9% vs 10%), females (8.9% vs 8.2%) and pregnant women (18.1% vs 7.3%) was higher in those screened by VHV compared to BHS (Table 2). As a result, a higher proportion of children aged < 5 years (10.3% vs 7.5%) and children aged 5–14 years (23.2% vs 15.9%) and females (34.9% vs 31.5%) compared to BHS with malaria given access to further treatment services by VHV compared to BHS. The VHV (73.6%) diagnosed higher proportion of *P. falciparum* (± mixed with *P. vivax*) compared to BHS (66.2%) (Table 3).

#### Quality of malaria treatment services

The proportion of patients with malaria provided treatment was higher (p < 0.01) among those who received care from VHV (96.6%) compared to BHS (94.9%). Of those provided treatment, 44.7% patients were given treatment within 24 h of fever in VHV group compared to 34.1% in BHS group (p < 0.01). However, the provision of complete treatment was lower (p < 0.01) among patients who were treated by VHV (80.9%) compared to BHS (88.2%) (Table 3).

The unadjusted and adjusted analyses on factors associated with treatment initiation is given in Table 4. The chance of receipt of treatment was higher among children < 5 years [aPR (95% CI) 1.030 (1.026, 1.034)]. However, the chance of receiving treatment was lower [aPR (95% CI) 0.989 (0.985, 0.993)] lower among children aged < 5 years compared to patients aged ≥ 15 years. After adjustment for available confounding factors, the chance of receipt of treatment among patients with malaria diagnosed by VHV was higher than patients diagnosed by BHS [aPR (95% CI) 1.017 (1.015, 1.020)].

**Table 4:** Demographic, clinical and treatment characteristics of patients with malaria associated with receipt of treatment in Myanmar, 2015

Characteristics	Total	Patients received treatment	Unadjusted prevalence ratio (95% CI)^e^	Adjusted prevalence ratio (95% CI)
	n (%)^a^	n (%)^b^		
Total	107,617 (100)	102,643 (95.4)		
Age (years)				
< 5	8917 (8.3)	8759 (98.2)	1.031 (1.027–1.034)	1.030 (1.026–1.034)
5–14	19,324 (18.0)	18,234 (94.4)	0.990 (0.986–0.994)	0.989 (0.985–0.993)
≥ 15	79,376 (73.8)	75,650 (95.3)	1	1
Sex				
Female	34,901 (32.4)	33,164 (95.0)	0.995 (0.992–0.997)	0.993 (0.990–0.996)
Male	72,716 (67.6)	69,479 (95.5)	1	1
Type of health worker				
VHV	30,090 (28.0)	29,054 (96.6)	1.017 (1.015–1.020)	1.017 (1.015–1.020)
BHS	77,527 (72.0)	73,589 (94.9)	1	
Malaria species^c^				
Pf	70,256 (65.3)	67,060 (95.5)	0.999 (0.997–1.002)^f^	Not included in the model
Pv	34,145 (31.7)	32,612 (95.5)	1	
Mixed	3210 (3.0)	2968 (92.5)	0.968 (0.958–0.978)	
Severity^d^				
Complicated	1340 (1.3)	1058 (79.0)	0.823 (0.800–0.846)	Not included in the model
Uncomplicated	101,999 (98.7)	97,885 (96.0)	1	

VHV: Village health volunteers, BHS: Basic health staff; CI: confidence interval; Pf: *Plasmodium falciparum*; Pv: *Plasmodium vivax*; Mixed: Both Pf and Pv positive

^a^ Column percentage; ^b^ Row percentage; ^c^ 6 Plasmodium malariae and ovale cases omitted from the analysis; ^d^ severity missing in 4278 cases; ^e^ p < 0.05 in all cases; ^f^ p—0.664

## Discussion

The current study assessed the performance of VHV compared to BHS in terms of providing access and quality of malaria care to patients diagnosed in 2015 under NMCP of Myanmar. In both VHV and BHS groups, the proportion of patients diagnosed with malaria among screened was similar. However, higher proportion of children (< 15 years) and women with undifferentiated fever were provided malaria-screening services by VHV compared to BHS. Similarly, the chance of receipt of treatment and treatment initiation within 24 h was statistically significantly higher among patients who were diagnosed by VHV compared to BHS after adjustment to potential confounders. However, the receipt of complete treatment was significantly lower among patients treated by VHV than BHS.

This was a countrywide study with a large sample, including all patients with undifferentiated fever screened and treated under NMCP. The study is based on routine programme data, showing the ground realities of the national programme. Usually the performance of VHV is assessed using fixed programme cut-offs for different indicators. To the best of the authors’ knowledge, this is one of the first studies to compare the performance of VHV with BHS in providing malaria screening and treatment services in Myanmar using routine programme data. Although there was a cluster randomized controlled trial done in Bago region of Myanmar (hereafter known as Bago RCT), the primary objective was basically to assess the feasibility of placing VHV in villages to improve coverage of malaria screening and treatment services to reduce malaria mortality [12].

The current study has shown that a significantly higher number and proportion of patients with fever were screened for malaria by BHS compared to VHV. This is very similar to Bago RCT, where BHS (midwives) screened a higher proportion (63%) of patients compared to VHV (37%) as BHS outnumber VHV. This high number and proportion of malaria screening among the population by BHS may also be due to high population coverage (nearly 70% population), and long duration of availability in health system [12]. According to the new NMTG, all fever cases should be suspected as malaria and tested with RDT [25]. BHS may also have the additional opportunity to screen the population at health facility/community level when delivering services related to other national programmes, especially maternal and child health programme. Similarly, VHV role is to complement the formal health system and provide malaria care services only in rural, remote and hard to reach areas.

The lower proportion of screening among children aged < 5 years by VHV could be due to low burden of fever in the community and direct referral of all fever cases to higher health facility as per NMTG. This may be due to higher access of children aged < 5 years by BHS to deliver other health services like immunization and nutrition.

A higher proportion of children aged < 15 years and females were provided access to RDT by VHV in this study, similar to Bago RCT. This could be due to VHV: (a) immediately available in the village (less travel); (b) established good rapport/trust with the community; (c) active case detection by house-to-house survey; (d) more affordable; (e) socially and culturally acceptable; (f) immediate availability of RDT [12, 26]. The acceptability of VHV remained high across countries irrespective of delivery of malaria screening or treatment services, either stand-alone as in Myanmar or integrated with other health interventions such as integrated management of childhood diseases as in Burkino Faso, Nigeria and Uganda [26].

A similar proportion of malaria positivity among VHV and BHS groups indicates the risk group identification for malaria screening was same. However, the high malaria positivity among < 5, 5–14 years, females and pregnant women may be due to more active case finding by VHV at community level and opportunistic/passive screening by BHS at health facility. Similarly, the *P. falciparum* positivity was also higher in the VHV group compared to BHS. Zero incidence of *P. falciparum* and mortality is an important step towards malaria elimination in the country [1]. To achieve the same, early detection and prompt treatment of all cases of falciparum malaria is needed to prevent transmission and death.

The proportion of malaria patients provided treatment by VHV (96.6%) in the current study is comparable to studies done in West Africa (90%) and the summary effect reported from a systematic review (97.7%) [26, 27]. The World Health Organization fixed a target for providing malaria testing and treatment initiation to at least 60% patients within 24 h of onset of fever/symptom [28]. Data on timing of malaria testing from the onset of fever, which is one of the important indicator for early detection of cases, was not available in the current study. However, the current study reported that 37.2% (44.7% in VHV and 34.1% in BHS) of patients with fever were tested and initiated treatment within 24 h of fever, which is one of the important achievements over a period of time in Myanmar compared to Bago RCT in 2012. The reported median (1st and 3rd quartile) duration for malaria test from onset of fever in the Bago RCT was 4 (2, 6) days and 3 (2, 4) days in VHV and midwives groups respectively [12]. However, Ghana, Uganda and Nigeria reported 90% achievement in providing treatment within 24 h of fever among children aged < 5 years [26].

Proportion of patients not initiated on treatment was higher among patients with complicated malaria (especially in VHV group) could be due to referral of all complicated malaria cases to higher health facility with or without first dose of anti-malarial drugs. Similarly, low proportion of patients aged 5–14 years were initiated on treatment. This may be due to neglect of this age group due to more importance given to under five age group and non-availability of patients as this age group is primarily a school going population. In addition, all pregnant women and children aged ≤ 1 year are supposed to be referred to higher health facility as per the guidelines which may reduced the proportion received the treatment.

Although VHV provided treatment to a higher proportion of patients, fewer of the patients received complete treatment compared to BHS. The reasons for high proportion of incomplete or inappropriate treatment could be due to different schedule of providing primaquine in VHV group (weekly once for 8 weeks) compared to BHS (daily for 14 days) in case of *P*. *vivax* and also be due to referral of complicated cases, pregnant women, and children aged ≤ 1 year to higher health facility with or without first dose of anti-malarial drugs.

The study had few limitations. Only the NMCP VHV data were analyzed since national and international NGO data were available only as aggregate data. The statistical significance observed in this study should be interpreted with caution for public health significance and practical application as the study dealt with big data which shown statistical significance even with small difference in proportion between groups. As the study was based on secondary data entered in malaria registers, the quality of data must be improved as there were missing data on severity of malaria [4% (5.5% in VHV and 3.4% in BHS)] and time of treatment initiation [14.1% (11.3% in VHV and 15.2% in BHS)], which were not included in the analysis. Complicated malaria reporting was very low (1%) which needs further review since there may be under-reporting. The study was able to assess only delivery of complete treatment and did not include follow up data on adherence to medication by the patients and status of clinical outcome at the end of treatment, which needs further research. The diagnosis and treatment outcome of patients with negative malaria test is not available as these patients are referred to BHS by VHV routinely and no data available on the same. However, it will be collected in future when VHV role is upgraded as integrated community malaria volunteers. Assessment of stock outs and other inventory difficulties were not assessed as the data was not available which needs comprehensive assessment in future. Linkage with routine surveillance system needed outbreak respose if any and further containment of transmissions.

## Recommendations

Modular training on malaria diagnosis and treatment can be provided at regular intervals to both VHV and BHS for improving and sustaining the access and quality of malaria care, especially improving the delivery of complete treatment. Use of information and communication technologies, such as mobile phones or other electronic devices, may be considered for collection of routine programmatic data for better and real time monitoring and surveillance. Regular evaluation of VHV and BHS activities must be carried out by the national programme to assess the performance and address the operational issues and barriers in delivering services. Appointment of more VHV in Myanmar, and standardization of services by developing clear operating procedures may increase efficiency of the services. This study also warrants a future qualitative study to explore and address the challenges faced by VHV in screening and providing treatment, especially in providing complete treatment. Further research is required on cost effectiveness of integrating VHV in malaria or other diseases control interventions.

## Conclusion

VHV were more accessible to children and women than BHS in providing malaria screening services. Malaria treatment services provided by VHV were as good as BHS. It is recommended to continue and recruit more VHV to provide community-based malaria care and assist the country towards malaria elimination. Further qualitative research to explore and address the challenges on initiation and delivering complete treatment by VHV including inventory assessment and cost-effectiveness studies on integration of VHV in routine health system are needed.
